# Plant Extract and Essential Oil Application against Food-Borne Pathogens in Raw Pork Meat

**DOI:** 10.3390/foods11060861

**Published:** 2022-03-18

**Authors:** Ioanna Mantzourani, Maria Daoutidou, Marilena Dasenaki, Anastasios Nikolaou, Athanasios Alexopoulos, Antonia Terpou, Nikolaos Thomaidis, Stavros Plessas

**Affiliations:** 1 Laboratory of Food Processing, Faculty of Agriculture Development, Democritus University of Thrace, 68200 Orestiada, Greece; maridaou3@agro.duth.gr (M.D.); anikol@mbg.duth.gr (A.N.); splessas@agro.duth.gr (S.P.); 2 Department of Chemistry, National and Kapodistrian University of Athens, Panepistimiopolis Zographou, 15771 Athens, Greece; mdasenaki@chem.uoa.gr (M.D.); ntho@chem.uoa.gr (N.T.); 3 Laboratory of Microbiology, Biotechnology & Hygiene, Faculty of Agriculture Development, Democritus University of Thrace, 68200 Orestiada, Greece; alexopo@agro.duth.gr; 4 Department of Agricultural Development, Agri-food, and Natural Resources Management, School of Agricultural Development, Nutrition & Sustainability, National and Kapodistrian University of Athens, GR-34400 Psachna, Greece; aterpou@agro.uoa.gr

**Keywords:** food-borne pathogens, plant extracts, pork meat, essential oils, antimicrobial activity, antioxidant activity

## Abstract

Herbal and plant extracts are being applied for a wide range of foods against different types of food-borne pathogens. In the present study, ethanolic and aqueous extracts (2% *w*/*v*) from cranberry (*Vaccinium macrocarpon*) and pomegranate (*Punica granatum* L.) plants were applied alone or in combination with two essential oils (thyme and oregano in a concentration of 0.150 μg/g) in pork meatballs and their antimicrobial activity was estimated. The extracts exhibited promising results (aqueous and ethanolic extracts of pomegranate and cranberry in a food-compatible concentration of 2% *w*/*v*) were applied to raw pork meatball production and their antimicrobial activity was recorded versus *Enterobacteriaceae*, total mesophilic bacteria, yeasts/molds, *Staphylococcus* spp., *Pseudomonas* spp. and lactic acid bacteria (LAB). The outcome demonstrated that meatballs containing aqueous extracts of pomegranate were more resistant to spoilage compared to all the other samples since they were preserved for more days. The chemical profiles of plant extracts were determined through LC-QTOF/MS and the chemical composition of the essential oils applied was determined with the use of GC/MS in order to identify the substances involved in the observed antimicrobial activity. Phenolic acids (quinic acid, chlorogenic acid), monoterpenes (*p*-cymene, carvacrol, thymol, limonene), organic acids (citric acid) and phenols were the main constituents found in the plant extracts and essential oils applied. These extracts of plant origin could be used as natural preservatives in meat products, even in low concentrations.

## 1. Introduction

Pork meat is one of the most susceptible foods for lipid oxidation, especially in minced form. Along with lipid oxidation, microbial contamination is the main cause of meat deterioration. Moreover, meat and its products are common sources of food-borne pathogens, namely *Escherichia coli*, *Staphylococcus aureus*, *Salmonella* spp. and *Listeria* spp. [1].

These factors may lead to off-odor and off-flavor development, discoloration, changes in texture and decreased shelf-life [2]. All these alterations affect consumers’ will to buy the meat products that exhibit them and cause economic losses to the meat industry [3].

Products resulting from peroxidation of polysaturated fatty acids (PUFA) and/or possessing increased microbial counts pose a risk to human health, increasing the frequencies of tumors and atherosclerosis [4].

At this exact point, the use of antioxidants is one of the main strategies in order to confront lipid oxidation and microbial contamination in meat products. Synthetic antioxidants are applied with quite satisfying results, but even if they are characterized as GRAS (generally recognized as safe), their extensive use is restricted by legislation, mostly because of potential health risks [2,3].

In addition, during the latest years, consumers’ concern about the impact on health that food consumption may have has led to a demand for more natural foods and preservatives [5].

Plant extracts and essential oils (EOs) are among these alternatives. Among numerous plant origin extracts, cranberry (*Vaccinium macrocarpon*) and pomegranate (*Punica granatum* L.) are being used in the food industry, mostly because of their antibacterial and antifungal activity [6].

More specifically, cranberry belongs to the *Ericaceae* family and has been consumed for centuries mostly because of its medicinal properties and its activity against urinary tract infections, dental decay, stomach ulcers and its antiproliferative action [7]. Several compounds in cranberry, including phenolic acid, tannins, proanthocyanidins and flavonoids, are responsible for its medicinal activities [8].

Pomegranate is characterized by a high concentration of tannins, which are mostly responsible for its antimicrobial properties. The plant belongs to the *Punicaceae* family and its peel is used in traditional medicine as a remedy for diarrhea and dysentery [9].

According to the literature, it is known that extraction with different kind of solvents, polar or non-polar or even their combination, may provide phytochemicals (flavonols, hydroxy-cinnamic acid), which exhibit bacteriostatic or bactericidal activity against food-borne or pathogenic bacteria [10].

Thyme essential oil (TEO) and oregano essential oil (OEO) are widely used as preservatives in foods and both are recognized as GRAS by the Food and Drug Administration (FDA). They are contained in *Thymus vulgaris* and *Origanum vulgare*, respectively, which belong to the *Lamiaceae* family [11]. Thyme and oregano EOs contain high concentrations of phenolic compounds, such as thymol, carvacrol, *p*-cymene and *γ*-terpinene, which cause structural and functional damage to the bacterial cell membrane [12]. Their effectiveness as natural antioxidants and antimicrobials has been previously tested in meat products [13].

The presence of carvacrol and thymol in the composition of these EOs was supposed to be responsible for the decreased lipid peroxidation and radical formation and the extension of shelf-life. Specifically, both oregano and thyme EO display good antimicrobial activity against Gram-negative and Gram-positive bacteria, most of them are common spoilage bacteria or food-borne pathogens within food systems [12].

Plant EOs are generally more active against Gram-positive than Gram-negative bacteria [14], mostly because of their outer membrane, which may restrict the diffusion of hydrophobic compounds through its lipopolysaccharide covering. Combinations of EOs and other extracts of plant origin with high phenolic content have been assessed for synergistic activity at lower concentrations in order to obtain the best antimicrobial and antioxidant activity with the least undesirable impact on the organoleptic properties of food [15].

In the present study, the application of aqueous cranberry and pomegranate extracts (2% *w*/*v*) and the application of thyme and oregano EOs (0.150 μg/g), alone or in combination between them, was examined in pork meatballs in refrigerated storage (4 °C), as far as their antimicrobial activity was concerned, since various microbial groups were examined. The total phenolic content (TPC) and the identity of the phenolic substances of the plant extracts were determined. In addition, the composition of the applied essential oils was revealed through gas chromatography-mass spectrometry. The upper goal of our experiments was to examine whether or not these natural preservatives could replace their synthetic counterparts in pork meat with equivalent antimicrobial and antioxidant activity.

## 2. Materials and Methods

### 2.1. Raw Materials

Fresh cranberries (*Vaccinium macrocarpon*) and pomegranates (*Punica granatum* L.) purchased from a local market of north-eastern Greece (Orestiada, Evros) were applied in the form of aqueous extracts in order to study their impact against several microbial groups.

The oregano essential oil (OEO) and the thyme essential oil (TEO) were commercially purchased from the local market of Nea Orestiada, Evros Regional Unit, Greece.

### 2.2. Preparation of Extracts

The extraction method applied was an adaptation from the study of Yang, Jia and Zu (2016) [16]. In brief, cranberry and pomegranate fruits were blended for approximately 60 s at 25 °C in a stomacher apparatus (Bagmixer, InterScience, SaintNomlaBretèche, France). Twenty grams of each puree was mixed with 200 mL of water (10% *w*/*v*) at 60 °C in an Erlenmeyer flask with agitation for two hours.

A 0.45 mm filter paper under vacuum was used for the filtration of the above aqueous solutions and centrifugation of the filtrates took place at 5000× *g* (PALL, LifeSciences, Portsmouth, UK) for 20 min at room temperature. Evaporation of the supernatants until a final volume of 5 mL at 50 °C in an evaporator (Model R204B3, Senco Technology Ltd., Shanghai, China) was the next step. Finally, 5 mL of distilled water was added (initial concentration: 2 g/mL) to the previous concentrated solutions. The samples were kept at −4 °C.

### 2.3. Preparation of Raw Meatballs

Greek traditional techniques were used for the preparation of the meatballs studied. More specifically, a batch consisting of ground pork meat (2.0 kg), lard (0.5 kg), sodium chloride 1.5% *w*/*w* (3.0 g) and black pepper (0.2 g) was produced. Homogenization of the mixture took place and 6 different types of meatballs were prepared; namely (a) control samples (C) in which no extract was used, (b) cranberry aqueous extracts (WC) and (c) pomegranate aqueous extracts (WP) with a concentration of 5% *v*/*v* in cranberry and pomegranate, respectively, added (all in a concentration of 2% *w*/*v*), (d) oregano essential oil (OEO), (e) thyme essential oil (TEO) with a concentration of 0.150 μg/g meatball and (f) respective combinations between cranberry aqueous extracts and TEO and OEO (WC + OEO, WC + TEO, WC + OEO + TEO) and (g) respective combinations of pomegranate aqueous extracts with OEO and TEO (WP + OEO, WP + TEO, WP + OEO + TEO) in the above concentrations. Meatball samples were kept at 4 °C in polystyrene trays for the entire study period. The microbiological experiments were repeated three times for each sample and collection took place at various intervals (1st, 4th, 7th, 8th, 12th day). Data after the 8th day of storage are not shown since the spoilage of meatballs was obvious in odor and appearance.

### 2.4. Microbiological Analysis of Meatball Portions

Several microbial food-borne pathogens were studied, such as Enterobacteria, total mesophilic bacteria (TMB), yeasts and molds, *Staphylococcus* spp. and *Pseudomonas* spp. In addition, the levels of LAB were determined. Initially, 25 g portions of meatball samples were homogenized in 225 mL of sterilized 1/4 Ringers solution (Sigma-Aldrich, St. Louis, MO, USA) from which decimal dilutions were prepared in tubes with sterilized 9 mL ¼ Ringers. An amount of 0.1 mL of homogenate was spread-plated on the surface of the following media: plate count agar (Oxoid Ltd., Hampshire, UK) for the detection of total mesophilic counts, acidified MRS agar (Oxoid Ltd., Hampshire, UK) for LAB, violet red bile glucose agar (Oxoid Ltd., Hampshire, UK) for enterobacteria, pseudomonas CFC selective agar for pseudomonads, Baird-Parker egg yolk tellurite agar for staphylococci and acidified (pH 4.5) malt agar (Oxoid Ltd., Hampshire, UK) for yeast and molds. Incubation periods varied from 24, 48 and 72 h according to the medium and temperature kept at 30 °C for TMC, pseudomonads and yeast/molds or at 37 °C for LAB, enterobacteria and staphylococci. A representative number of isolated colonies were Gram-stained and tested for the presence of catalase, particularly for LAB confirmation.

### 2.5. Determination of Total Phenolic Content (TPC)

The total phenolic content of aqueous cranberry and pomegranate extracts was determined according to A. Waterhouse’s method [17]. More specifically, a calibration curve was prepared with solutions of gallic acid diluted in ethanol and afterwards in water. The concentrations varied from 0–1000 mg/L gallic acid.

After the preparation of the calibration curve, 20 μL from each extract was pipetted into cuvette with 1.58 mL water and 100 μL of Folin-Ciocalteau reagent and mixed well. After 5 min, 300 μL of sodium carbonate solution (20% *w*/*v*) was added. The solutions were left at 20 °C for 2 h and the absorbance of each solution was determined at 765 nm. Results are reported as gallic acid equivalent (GAE).

### 2.6. GC/MS Analysis

An amount of 1 mL of each essential oil (OEO and TEO) was diluted with 9 mL of pure hexane and filtrated using 0.22 μm filters. The mixtures’ chemical composition was determined by GC-MS analysis (6890N GC, 5973NetworkedMS MSD (Agilent Technologies, Santa Clara, CA, USA)) as previously described [18], with some modifications. In brief, 1 μL of each sample was directly injected into an HP-5MS column (30 m, 0.25 mm i.d., 0.25 μm film thickness). Carrier gas (He) flow was 1.5 mL/min in split mode (1:50). The injector and the detector temperatures were set at 240 °C. Oven temperature was set at 35 °C, held constant for 6 min, followed by an increase to 60 °C at a 2 °C/min rate and held constant for 5 min. A new increase to 200 °C at a 5 °C/min rate was followed by a final increase to 250 °C at a 25 °C/min rate, held constant for 5 min. The mass spectrometer was operated in the electron impact mode (electron energy at 70 eV and scan range at 45 to 400 *m*/*z*). Results acquired were processed by ChemStation integrated software (Agilent Technologies), and compounds were identified by comparing the retention times and mass spectra to NBS75K and Wiley275 reference libraries and in-house libraries, and by determining Kovats retention indexes (KIs). Both samples’ analyses were carried out in triplicate and the mean data are presented (standard deviation for all values was about ±5%).

### 2.7. LC-QTOF/MS Analysis

The determination of target analytes was performed using an ultrahigh-performance liquid chromatography (UHPLC) system with an HPG-3400 pump (Dionex Ultimate 3000 RSLC, Thermo Fischer Scientific, Dreieich, Germany) coupled to a QTOF mass spectrometer (Maxis Impact, Bruker Daltonics, Bremen, Germany). The method has been described previously [19]. QTOF external calibration was realized every day using sodium formate in a mixture of isopropanol:water (50:50 *v*/*v*). Moreover, internal calibration was performed by calibrant injection starting each run (1st segment, 0.1–0.25 min). There was a typical resolving power (full-width at half-maximum, FWHM) between 35,000 and 40,000 at *m*/*z* 226.1593 of 430.91.

### 2.8. Statistical Analysis

Bacterial populations in ground pork meat are presented as mean ± standard deviation of Log CFU/g. Bacterial counts between various treatments (fruit extracts) and also between consecutive days (in each species) were estimated by using the ANOVA procedure with Tukey’s HSD post hoc comparisons. Statistical analysis was performed with SPSS v20^®^ (IBM Corp. Armonk, NY, USA) at a 95% significance level.

## 3. Results

### 3.1. Microbiological Analyses

The bacterial counts in ground beef meatballs during storage (4 °C/7 days) treated with various combinations of pomegranate and cranberry aqueous extract (1% *w*/*v*) as well as oregano and thyme essential oils (0.150 μg/g of meatball) are presented in Table 1. The respective zones of inhibition (ZOIs) measured (in mm) are included.

*Enterobacteriaceae* were abundant in almost all of our samples and average populations ranged from undetected to 6.79 Log CFU/g. In control samples *Enterobacteriaceae* were found in a mean population of 3.09 ± 0.5 Log CFU/g during the first day and up to 7.49 ± 0.4 Log CFU/g after 7 days. Even from the first day, all treatments statistically differed from the control regarding the counts of *Enterobacteriaceae* (ANOVA F = 10.06, *p* < 0.05) indicating an immediate effect of their antimicrobial potential against this particular group of pathogens.

**Figure 1:**
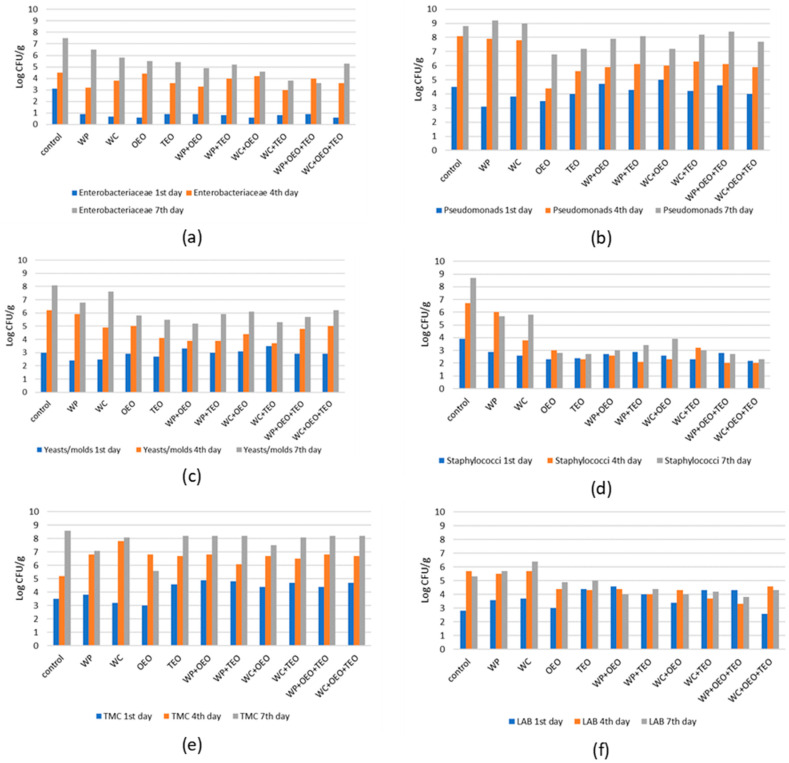
Total counts of (**a**) *Enterobacteriaceae*, (**b**) *Pseudomonas* spp., (**c**) yeasts and molds, (**d**) *Staphylococcus* spp., (**e**) total mesophilic counts (TMCs), and (**f**) LAB detected on meatballs after 1, 4 and 7 days of storage at 4 °C.

However, this effectiveness was not observed during the 4th day of storage (ANOVA F = 2.14, *p* > 0.05) but reappeared by the end of the observation period on the 7th day (ANOVA F = 18.43, *p* < 0.05). The combination of oregano EO, thyme EO and pomegranate aqueous extract resulted in the most notable delay of *Enterobacteriaceae* growth (Figure 1a).

*Pseudomonads* were also recovered from all samples. Mean control counts during the 1st day were 4.49 ± 0.3 Log CFU/g and 8.79 ± 0.3 Log CFU/g on the 7th day, while in all other treatments counts ranged from minus 2 Log to plus 0.5 Log of the above value. Although some statistically significant differences during the consequent days of storage were observed among the various treatments, the lowest counts were found when the oregano EO was solely used (Figure 1b).

Yeasts and molds were the bacterial group with the highest increase (over 6 Logs) in our samples during the 7 days of observation. Mean control counts were initially 2.99 ± 0.1 Log CFU/g and rose to 8.09 ± 0.5 Log CFU/g on the last day. Significant differences during the study period were observed between the control counts and all treatments, except those with pomegranate or cranberry aqueous extracts (Figure 1c).

*Staphylococcus* spp. in our samples was counted from 2.29 ± 0.1 Log CFU/g to 8.69 ± 0.6 Log CFU/g. During the 1st day, the lowest bacterial populations were observed in the treatment with oregano EO (average 2.29 ± 0.1 Log CFU/g) and the one with thyme EO (TEO) combined with cranberry aqueous extract (WC + TEO). Both of these counts differed statistically from those of the control group (ANOVA F = 1.76, *p* < 0.05). However, during the 7th day, all treatments were statistically different from the control regarding the *Staphylococcus* spp. population (ANOVA, F = 33.56, *p* < 0.05), indicating a strong antibacterial action of the various extracts and essential oils, which appeared to be stronger when combinations of the above were used, lowering the final counts up to 6.4 Logs (as in the case of WC + OEO + TEO with an average 2.29 ± 0.4 Log CFU/g) (Figure 1d).

In all treatments, total mesophilic counts ranged from an average of 4.25 ± 0.7 Log CFU/g during the 1st day (control 3.5 ± 0.7 Log CFU/g) to 7.74 ± 0.8 Log CFU/g by the end of the study period (control 8.6 ± 0.3 Log CFU/g). There was no particular antimicrobial effectiveness of essential oils or fruit extracts against that microbial group besides the one observed for oregano EO during the 7th day of analysis, when 5.59 ± 0.8 Log CFU/g was observed in comparison with 8.59 ± 0.3 Log CFU/g for the control group (Figure 1e).

As shown in Table 1, initially (1st day) LABs were recovered in populations from 2.59 ± 0.7 Log CFU/g in WC + OEO + TEO samples to 4.59 ± 0.2 Log CFU/g in WP + OEO samples and reached as high as 6.39 ± 0.2 Log CFU/g in WC samples during the 7th day. During the observation period LABs showed a similar growth pattern in all treatments with the exception of the WP + OEO + TEO samples where their final counts were statistically lower than others (3.79 ± 0.7 Log CFU/g, ANOVA F = 9.82, *p* < 0.05) (Figure 1f).

### 3.2. Total Phenolic Content (TPC)

From the above analytical results (Table 2), it is obvious that the aqueous cranberry extract exhibits three-fold phenolic content in comparison with the respective pomegranate extract. This fact was verified by the following chemical profile of the extracts with the LC-QTOF/MS analysis, where the quantity and the number of phenolic compounds in the cranberry extract definitely increased.

### 3.3. Determination of Chemical Composition of Oregano and Thyme Essential Oils by GC/MS

Table 3 presents the chemical profile of the two essential oils used, thyme and oregano.

From the above, it becomes obvious that the main components of the oregano essential oil (OEO) applied are *p*-cymene (29.4%), carvacrol (26.6%), thymol (12.7%) and limonene (9.3%).

As far as thyme essential oil (TEO) is concerned, its chemical profile mostly consists of thymol (29.8%), *p*-cymene (27.3%), limonene (9.5%) and carvacrol (6.4%).

Other substances such as *a*-pinene, linalool, *a*-terpineol, *γ*-terpineol, caryophyllene and caryoplyllene oxide were detected in lower percentages ranging from 1.1% to 3.5% in both essential oils.

In OEO 24 compounds were determined, which constitute 92% of the volatile oil, and in TEO 23 compounds were detected, corresponding to 97.7% of the volatile oil.

The analysis revealed that in both essential oils, monoterpene hydrocarbons and oxygenated monoterpenes were the main group of chemicals.

### 3.4. Determination of Chemical Composition of Plant Extracts Using LC-QTOF/MS Analysis

From the above analytical results (Table 4), aqueous cranberry extract mainly consists of malic acid (96 mg/L) and citric acid (74.59 mg/L). Benzoic acid (3.21 mg/L) and chlorogenic acid (2.22 mg/L) follow. All the other components exist in quantities smaller than 0.3 mg/L and include substances such as *p*-coumaric acid (0.02 mg/L), quercetin (0.21 mg/L), hydroxytyrosol (0.04 mg/L), 4-hydroxybenzoic acid (0.03 mg/L) and galangin (0.02 mg/L).

Aqueous pomegranate extract consists mostly of citric acid (109.02 mg/L) and malic acid (28 mg/L) as major components. Several substances like galangin, quercetin, kaempferide, protocatechuic acid and kaempferol exist in this extract as in the aqueous cranberry extract. Nevertheless, components like 4-hydroxybenzoic acid, benzoic acid and hydroxytyrosol in this case are absent.

## 4. Discussion

Fundamentally, the antimicrobial mode of action of polyphenolic rich plant extracts includes the following:

(a) Molecules that disrupt the cell membrane, like the OH-group. These molecules induce increased membrane permeability and consequently leakage of cell content.

(b) Decreased pH of the medium because of the accumulated proton concentration and finally induced depolarization impacting the proton motive force leading to cell death.

(c) Organic acids that impact on the NADH oxidation [20].

Recent research concerning the antimicrobial activity of plant extracts demonstrated that the number of OH-groups of the phenolic ring plays important role in the observed activity [21]. The position of the OH-group on the aromatic ring is of equal importance. Research data reveal that meta-thesis is more active compared to ortho-thesis [22] and OH-groups in position 2′ of chalcones and position 5’ of flavanones and flavones increase their antimicrobial properties probably by the delocalization of electrons from the cytoplasmic membrane [23].

In addition, the presence of alkyl groups in the aromatic nucleus has been reported to interfere in the antimicrobial activity of plant-derived phenols and affect the distribution ratio between polar and non-polar phases, bacterial phases included [24].

As a general rule, it can be noted that Gram-negative bacteria are more resistant to phenolics than Gram-positive bacteria [25]. This is probably because of the cell wall that they possess and it is linked to the polysaccharide envelope, which prevents the entrance of phytochemicals [26].

A similar mode of antimicrobial and antioxidant activity was observed for essential oils. Although a certain amount of leakage from bacterial cells may not result in loss of viability, an extensive loss of cytoplasmic content will lead to cellular death [27]. Disturbance of the cytoplasmic membrane or disrupting the proton motive force are two other pathways of activity. The importance of the presence of OH-groups in phenolic compounds such as carvacrol and thymol is already known [23,28]. Other antimicrobial pathways of EO components include binding on membrane proteins, stimulation of pseudomycelia formation, inhibition of certain necessary metabolic enzymes, etc. [14].

Furthermore, these observations were also verified in our study. The growth of *Enterobacteriaceae* seemed to be inhibited more extensively when aqueous pomegranate extract combined with oregano essential oil (OEO) and thyme essential oil (TEO) was applied to the pork meat. From the chemical profile of the aqueous pomegranate extract, it can be concluded that citric and malic acid, two classic organic acids, are its major components. Their presence leads to a reduction in the pH value, creating an unpleasant environment for Enterobacteria and gradually leading them to death, depression of the internal pH of microbial cells by ionization of undissociated acid molecules and disruption of substrate transport by altering cell membrane permeability or reduction of the proton motive force [29].

In addition to the acidic conditions, both EOs with major components *p*-cymene, carvacrol and thymol appear to induce cell membrane permeability [30] and simultaneously disintegrate the outer membrane of Gram-negative bacteria, releasing lipopolysaccharides (LPSs) [31].

In general, the antimicrobial activity of these two EOs increases when the pH decreases. The inhibitory effect of plant extracts becomes greater at acidic pH values. *Pseudomonas* spp. seemed to be inhibited mostly when OEO was applied solely. Strains of this family are known to show consistently high resistance to plants’ antimicrobial activity [32]. Limonene has also been found to be more active than *p*-cymene [23]. Essential oils from different oregano species are known to exhibit high levels of antimicrobial activity against *Pseudomonas* strains. The fact that Pseudomonas genera do not exhibit the ability to tolerate the conditions generated by the presence of OEO may be associated with a disturbance in the proteinosynthesis of several metabolic enzymes [33]. The necessary concentration levels could be lower than the MIC (minimum inhibitory concentration).

As far as yeasts/molds are concerned, our results are in accordance with data from the literature. Rasooli et al. [34] analyzed the effect of *A. niger* exposure to MIC levels of TEO and they revealed severe damage to the cell wall, cell membrane and cellular organelles. The mycelium exposed to TEO exhibited morphological changes in the hyphae, disruption in the plasma membrane and mitochondrial destruction.

The fact that the aqueous pomegranate extract in combination with OEO and the aqueous cranberry combined with TEO are the most effective treatments against yeasts in the meat system studied should be attributed to the synergistic interaction between organic acids (malic and citric acid) from the plant extracts and *p*-cymene, carvacrol and thymol from the essential oils applied. It has been suggested that some hydrophobic compounds present in plant extracts could change the permeability of the microbial membranes for cations modifying cell pH and affecting cell activity. Nevertheless, higher solubility does not always mean greater antimicrobial action. Bagheri-Gavkosh et al. [35] showed that plant extracts suppressed mycotoxin production by *A. parasiticus*. This inhibition was explained by the presence of flavonoid compounds such as *p*-coumaric acid and quercetin in plant extracts. These substances were detected in the essential oils and plant extracts applied in our study as well.

*Staphylococcus* strains were mostly inhibited by the combination of the aqueous cranberry extracts with OEO and TEO. From previous studies, it has been established that carvacrol and thymol have additive effects obtained by interactions between volatile oils [36]. These two substances are isomers, suggesting a similar mechanism of action, which includes functional and structural damage to the outer and inner membrane and membrane proteins. The additive antimicrobial effect of these two EOs against *S. aureus* strains has been already demonstrated in several studies [11,37], verifying our findings.

Total mesophilic bacteria (TMB) were found to be more efficiently controlled by OEO when used solely. This fact was confirmed other studies as well, implying that synergistic inhibitory effects on food-borne bacteria are present when plant extracts are applied [38]. Other researchers suggest that TMB in meat and meat products can be limited during refrigerated storage by plant extracts and EOs or a combination of both [24,38].

More specifically, *p*-cymene and *γ*-terpinene as constituents of OEO, along with carvacrol and thymol, induce the strongest antimicrobial effect and control the population of total mesophilic bacteria [36]. In another study [39], similar findings were resumed when TEO and beet juice were applied to meat sausage and total mesophiles were estimated in refrigerated storage. Then, partial inhibition of TMB was observed when TEO in the highest concentration tested (0.95% *w*/*v*) was applied, replaced by 50% by nitrite.

Finally, the population of lactic acid bacteria (LAB) was determined. It is known that the presence of LAB on meats usually ensures that shelf-life is prolonged. After the inhibition of aerobic spoilage bacteria, LAB may become the dominant bacteria group. The population of LAB reached the lowest values when aqueous pomegranate extract and both EOs were applied in the meat samples (3.8 Log CFU/g during the 7th day) compared to the control (5.3 Log CFU/g during the 7th day). Notably, in every application including pomegranate or cranberry extract combined with one or both EOs, the population of LAB natural microbiota decreased compared to the respective day of refrigerated storage. The only exception was the case where the two aqueous extracts were tested solely in the meat sample. This can be attributed to the relatively low pH, especially in the case of aqueous cranberry extract. These results are in agreement with Menezes et al. [40]. Aminzare et al. [41] confirmed that the combination of *Zataria multiflora* EO, a thyme-like medicinal plant from Iran, and grape seed extract, when applied in cooked sausage, caused a reduction of total mesophilic and psychrotrophic viable counts and LAB. This reduction was attributed to the synergistic effect between thymol and carvacrol. The same conclusions can be made in our treatments.

## 5. Conclusions

In summary, the present study examined the antimicrobial activity of aqueous pomegranate and cranberry extracts alone or in combination with oregano and thyme essential oils solely or simultaneously. The food system used was minced pork meatballs and the microbial groups tested were *Enterobacteriaceae*, total mesophilic bacteria, yeasts/molds, *Staphylococcus* spp., *Pseudomonas* spp. and lactic acid bacteria (LAB). All the microbial group populations were reduced after the application of particular EOs and/or plant extracts in each case. The chemical profile of plant extracts and TEO-OEO was determined in order to clarify the interactions between the organic acids, phenolic acids and phenols with monoterpenes and triterpenes.

On this basis, the combinations of plant-derived extracts and EOs could replace synthetic antioxidants, which are widely used in meat and meat-product preservation as antimicrobials and antioxidants. Further investigation should take place in order for more alternative combinations and their possible sensorial attributes to be determined.

## Figures and Tables

**Table 1:** Bacterial counts (Log CFU/g) in ground beef meatballs during storage (4 °C/7 days) treated with various combinations of pomegranate and cranberry aqueous extract (2% *w*/*v*) as well as oregano and thyme essential oils (0.150 μg/g of meatball).

Species	Day of Analysis	C (Control)	WP	WC	OEO	TEO	WP + OEO	WP + TEO	WC + OEO	WC + TEO	WP + OEO + TEO	WC + OEO + TEO
Entero.	1st	3.09 ± 0.5 ^a1^	0.89 ± 0.3 ^b1^	0.7 ± 0.6 ^b1^	0.6 ± 0.2 ^b1^	0.89 ± 0.2 ^b1^	0.89 ± 0.5 ^b1^	0.8 ± 0.1 ^b1^	0.6 ± 0.2 ^b1^	0.8 ± 0.3 ^b1^	0.89 ± 0.7 ^b1^	0.6 ± 0.1 ^b1^
	4th	4.49 ± 0.8 ^a1^	3.19 ± 0.7 ^a2^	3.79 ± 0.7 ^a2^	4.39 ± 0.6 ^a2^	3.59 ± 0.2 ^a2^	3.29 ± 0.1 ^a2^	3.99 ± 0.8 ^a2^	4.19 ± 0.5 ^a2^	2.99 ± 0.3 ^a2^	3.99 ± 0.6 ^a2^	3.59 ± 0.6 ^a2^
	7th	7.49 ± 0.4 ^a2^	6.49 ± 0.3 ^abc3^	5.79 ± 0.6 ^bc3^	5.49 ± 0.7 ^bc2^	5.39 ± 0.3 ^bc3^	4.89 ± 0.2 ^cd3^	5.19 ± 0.1 ^b3^	4.59 ± 0.3 ^cd2^	3.79 ± 0.7 ^d2^	3.59 ± 0.2 ^cd2^	5.29 ± 0.6 ^bc3^
Pseudo.	1st	4.49 ± 0.3 ^ab1^	3.09 ± 0.7 ^a1^	3.79 ± 0.3 ^ab1^	3.49 ± 0.1 ^ab1^	3.99 ± 0.7 ^ab1^	4.69 ± 0.6 ^b1^	4.29 ± 0.7 ^a1^	4.99 ± 0.2 ^b1^	4.19 ± 0.8 ^ab1^	4.59 ± 0.1 ^ab1^	3.99 ± 0.7 ^ab1^
	4th	8.09 ± 0.8 ^d2^	7.89 ± 0.1 ^cd2^	7.79 ± 0.3 ^cd2^	4.39 ± 0.5 ^a2^	5.59 ± 0.7 ^ab2^	5.89 ± 0.7 ^ab1^	6.09 ± 0.6 ^b2^	5.99 ± 0.8 ^ab12^	6.29 ± 0.1 ^bc2^	6.09 ± 0.1 ^b2^	5.89 ± 0.8 ^ab12^
	7th	8.79 ± 0.3 ^cd2^	9.19 ± 0.5 ^d3^	8.99 ± 0.5 ^cd3^	6.79 ± 0.2 ^a3^	7.19 ± 0.3 ^ab3^	7.89 ± 0.8 ^abcd2^	8.09 ± 0.3 ^abcd3^	7.19 ± 0.5 ^ab2^	8.19 ± 0.3 ^abcd3^	8.39 ± 0.4 ^bcd3^	7.69 ± 0.8 ^abc2^
Y/M	1st	2.99 ± 0.1 ^a1^	2.39 ± 0.3 ^a1^	2.49 ± 0.3 ^a1^	2.89 ± 0.2 ^a1^	2.69 ± 0.7 ^a1^	3.29 ± 0.4 ^a1^	2.99 ± 0.1 ^a1^	3.09 ± 0.5 ^a1^	3.49 ± 0.6 ^a1^	2.89 ± 0.4 ^a1^	2.89 ± 0.5 ^a1^
	4th	6.19 ± 0.6 ^d2^	5.89 ± 0.5 ^cd1^	4.89 ± 0.1 ^abc2^	4.99 ± 0.6 ^bcd2^	4.09 ± 0.3 ^ab2^	3.89 ± 0.1 ^ab2^	3.89 ± 0.3 ^ab1^	4.39 ± 0.6 ^a2^	3.69 ± 0.1 ^a1^	4.79 ± 0.3 ^abc2^	4.99 ± 0.6 ^bcd2^
	7th	8.09 ± 0.5 ^c3^	6.79 ± 0.8 ^abc2^	7.59 ± 0.5 ^bc3^	5.79 ± 0.7 ^a2^	5.49 ± 0.4 ^a3^	5.19 ± 0.3 ^a2^	5.89 ± 0.8 ^a2^	6.09 ± 0.3 ^ab3^	5.29 ± 0.2 ^a2^	5.69 ± 0.3 ^a3^	6.19 ± 0.8 ^ab2^
Staph.	1st	3.89 ± 0.5 ^b1^	2.89 ± 0.3 ^ab1^	2.59 ± 0.1 ^ab1^	2.29 ± 0.7 ^a1^	2.39 ± 0.4 ^ab1^	2.69 ± 0.2 ^ab1^	2.89 ± 0.8 ^ab1^	2.59 ± 0.2 ^ab1^	2.29 ± 0.5 ^a1^	2.79 ± 0.7 ^ab1^	2.19 ± 0.8 ^a1^
	4th	6.69 ± 0.4 ^c2^	5.99 ± 0.7 ^c2^	3.79 ± 0.6 ^b1^	2.99 ± 0.3 ^ab1^	2.29 ± 0.3 ^a1^	2.59 ± 0.2 ^ab1^	2.09 ± 0.3 ^a1^	2.29 ± 0.8 ^a12^	3.19 ± 0.5 ^ab1^	1.99 ± 0.8 ^a1^	1.99 ± 0.1 ^a1^
	7th	8.69 ± 0.6 ^c3^	5.69 ± 0.7 ^b2^	5.79 ± 0.8 ^b2^	2.79 ± 0.3 ^a1^	2.69 ± 0.3 ^a1^	2.99 ± 0.1 ^a1^	3.39 ± 0.8 ^a1^	3.89 ± 0.4 ^a2^	2.99 ± 0.7 ^a1^	2.69 ± 0.8 ^a1^	2.29 ± 0.4 ^a1^
TMC	1st	3.49 ± 0.7 ^ab1^	3.79 ± 0.6 ^ab1^	3.19 ± 0.8 ^ab1^	2.99 ± 0.5 ^a1^	4.59 ± 0.3 ^ab1^	4.89 ± 0.8 ^b1^	4.79 ± 0.1 ^b1^	4.39 ± 0.5 ^ab1^	4.69 ± 0.4 ^ab1^	4.39 ± 0.8 ^ab1^	4.69 ± 0.6 ^ab1^
	4th	5.19 ± 0.8 ^a2^	6.79 ± 0.6 ^ab2^	7.79 ± 0.4 ^c2^	6.79 ± 0.1 ^ab2^	6.69 ± 0.1 ^bc2^	6.79 ± 0.6 ^bc2^	6.09 ± 0.6 ^ab2^	6.69 ± 0.1 ^bc2^	6.49 ± 0.7 ^abc2^	6.79 ± 0.2 ^bc2^	6.69 ± 0.4 ^bc2^
	7th	8.59 ± 0.3 ^b3^	7.09 ± 0.5 ^ab2^	8.09 ± 0.8 ^b2^	5.59 ± 0.8 ^a2^	8.19 ± 0.6 ^b3^	8.19 ± 0.6 ^b2^	8.19 ± 0.1 ^b3^	7.49 ± 0.3 ^b2^	8.09 ± 0.3 ^b3^	8.19 ± 0.8 ^b3^	8.19 ± 0.6 ^b3^
LAB	1st	2.79 ± 0.1 ^ab1^	3.59 ± 0.8 ^abcd1^	3.69 ± 0.6 ^abcd1^	2.99 ± 0.7 ^abc1^	4.39 ± 0.5 ^cd1^	4.59 ± 0.2 ^d1^	3.99 ± 0.5 ^abcd1^	3.39 ± 0.3 ^abcd1^	4.29 ± 0.1 ^bcd1^	4.29 ± 0.6 ^bcd1^	2.59 ± 0.7 ^a1^
	4th	5.69 ± 0.6 ^ab2^	5.49 ± 0.1 ^abcd2^	5.69 ± 0.2 ^abcd2^	4.39 ± 0.7 ^abc12^	4.29 ± 0.1 ^cd2^	4.39 ± 0.2 ^d12^	3.99 ± 0.3 ^abcd2^	4.29 ± 0.1 ^abcd2^	3.69 ± 0.2 ^bcd1^	3.29 ± 0.8 ^bcd1^	4.59 ± 0.6 ^a2^
	7th	5.29 ± 0.5 ^bcd2^	5.69 ± 0.6 ^cd2^	6.39 ± 0.2 ^d2^	4.89 ± 0.5 ^abc2^	4.99 ± 0.5 ^abc3^	3.99 ± 0.2 ^ab2^	4.39 ± 0.4 ^abc3^	3.99 ± 0.2 ^ab1^	4.19 ± 0.6 ^ab1^	3.79 ± 0.7 ^a1^	4.29 ± 0.1 ^ab2^

Different superscript letters in rows indicate statistically significant differences of the various bacterial counts between treatments (WP, WC, OEO, TEO, WP + OEO, WP + TEO, WC + OEO, WC + TEO, WP + OEO + TEO, WC + OEO + TEO). Different superscript numbers in columns indicate statistically significant differences in the various bacterial counts during consecutive days of analysis (i.e., 1st, 4th and 7th).

**Table 2:** Total phenolic content of the aqueous pomegranate and cranberry extracts expressed as mg/mL of gallic acid.

	mg/mL GA
Aqueous Pomegranate Extract	51.70
Aqueous Cranberry Extract	185.85

**Table 3:** Chemical profile of the two essential oils used, oregano (OEO) and thyme (TEO).

Compounds Detected	ΚΙ	% Area OEO	% Area ΤΕO
methyl-Cyclopentane	<800	0.1	1.0
α-Pinene	928	3.2	3.2
Camphene	943	0.4	0.1
Benzaldehyde	964	n.d.	0.1
*β*-Pinene	975	0.1	0.8
*β*-Myrcene	1001	0.1	0.1
3-Carene	1016	n.d.	2.9
α-Terpinene	1026	0.3	n.d.
*p*-Cymene	1044	29.4	27.3
Limonene	1046	9.3	9.5
*p*-Cymenene	1126	0.1	n.d.
α-Terpinolene	1122	0.6	n.d.
Linalool	1143	2.2	1.1
1-Terpinenol	1167	0.2	0.3
Borneol	1189	0.8	3.5
Isoborneol	1181	0.1	0.7
Terpinen-4-ol	1195	0.2	0.2
α-Terpineol	1214	3.4	3.1
*β*-Terpineol	1176	0.6	0.4
*γ*-Terpineol	1220	1.2	1.1
Citronellol	1258	0.1	n.d.
Linalyl acetate	1280	0.1	1.8
Thymol	1345	12.7	29.8
Carvacrol	1357	26.6	6.4
Caryophyllene	1442	0.1	2.9
α-Caryophyllene	1479	n.d.	0.3
Caryophyllene oxide	1612	0.1	1.1
Total		92.0	97.7

n.d.: not detected; KI: Kovats retention index.

**Table 4:** Chemical composition of aqueous extracts of cranberry and pomegranate expressed in mg/L.

Compounds Detected	Aqueous Cranberry Extract	Aqueous Pomegranate Extract
4-hydroxybenzoic acid	0.03	n.d.
Galangin	0.02	0.02
Malic acid	96.00	28.00
Quercetin	0.21	0.30
Kaempferide	0.03	0.04
Catechol	0.01	0.01
Apigenin	0.01	0.01
3,4-Dihydroxybenzoic acid (protocatechuic acid)	0.07	0.05
Luteolin	0.07	0.10
Acacetin	0.005	0.01
Benzoic acid	3.21	n.d.
Kaempferol	0.06	0.09
Chlorogenic acid	2.22	<0.01
*p*-coumaric acid	0.68	0.07
Citric acid	74.59	109.02
Hydroxytyrosol	0.04	n.d.
Chrysin	<0.01	<0.01
Quinic acid	75.00	0.03

## Data Availability

Data is contained within the article.
